# Genome-wide CRISPR knockout screen identifies activating transcription factor (ATF1) as an activator of HIV gene expression

**DOI:** 10.1128/mbio.00557-25

**Published:** 2025-07-03

**Authors:** Alona Kuzmina, Seraj Wattad, Praveenkumar Murugavelu, Noa Amir, Nili Tickotsky, Liron Levin, Ran Taube

**Affiliations:** 1 The Shraga Segal Department of Microbiology Immunology and Genetics, Faculty of Health Sciences, Ben-Gurion University of the Negev 85456 , Be'er Sheva, Israel; 2 Bioinformatics Core Facility, Ilse Katz Institute for Nanoscale Science and Technology, Ben-Gurion University of the Negev 626088 , Be'er Sheva, Israel; Albert Einstein College of Medicine, Bronx, New York, USA

**Keywords:** activating factor (ATF1), HIV gene transcription regulation, latency, CCR5 co-receptor, CCR5 antisense lncRNA

## Abstract

Antiretroviral therapy against the human immunodeficiency virus (HIV) has significantly prolonged the life span of people living with HIV, transforming viral infection into a latent condition that is characterized with undetectable viral loads. Yet, a complete cure of infection is out of reach, as transcriptionally silent but replication-competent proviruses persist in a long-lived reservoir that is resistant to therapy. The current work follows a genome-wide CRISPR knockout screen in human CD4^+^ T cells and defines the activating transcription factor 1 (ATF1) as an activator of HIV gene transcription with elevated expression levels in cells that carry transcriptionally active provirus. Additional gain and loss-of-function experiments show that depletion of ATF1 promotes latency. ATF1 directly occupies the HIV promoter, where it regulates the recruitment of RNA Polymerase II and the levels of H3K9me3 histone repression mark. Genome-wide, ATF1 binds cellular gene promoters. Among its targets, ATF1 modulates the levels of CCR5 antisense lncRNA, thereby regulating the protein expression of the CCR5 HIV co-receptor. We conclude that ATF1 is an activator of gene transcription that dictates HIV gene expression via both direct and indirect mechanisms.

**IMPORTANCE:**

HIV persists in resting CD4^+^ primary infected cells, forming a reservoir that is resistant to therapy, and thus a main barrier toward elimination of viral infection. An understanding of the mechanisms that control HIV gene expression and drive viral latency is therefore of high clinical importance. This study identifies activating transcription factor 1 (ATF1) as an activator of HIV gene expression. ATF1 binds the HIV promoter, where it modulates the occupancy of RNA Polymerase II and the levels of H3K9me3 histone repression mark. Genome-wide, ATF1 also occupies cellular promoters. One target of ATF1 is the antisense (AS) lncRNA. Through binding to CCR5-AS lncRNA, ATF1 induces CCR5 mRNA stability, thereby indirectly controlling HIV infection. Overall, we provide an additional understanding of the host transcription pathways that regulate HIV gene expression and potentially open new ways to manipulate its reservoir size.

## INTRODUCTION

The implementation of antiretroviral therapy (ART) has effectively restricted the transmission of human immunodeficiency virus (HIV) and improved overall clinical outcomes. However, a complete cure for HIV remains out of reach, as the virus persists in a stable pool of infected cellular reservoirs, which are comprised of memory-resting CD4^+^ T cells, as well as cells of myeloid lineages. The infected cell reservoirs are long-lived, resistant to therapy and to the effects of the host immune surveillance—thus a main barrier toward complete elimination of viral infection (1, 2). Consequently, in most people living with HIV, interrupting ART results in a rapid rebound of viral load, usually within weeks after treatment cessation (3–6). As T-cell stimulation triggers activation of proviral transcription, one strategy that has been proposed to eliminate the HIV reservoirs is a “Shock-and-Kill” approach, which utilizes latency-reversing agents (LRAs) to first activate dormant HIV-infected T cells and facilitate cell death by viral cytopathic effects or immune-mediated killing. This step is conducted in the presence of ART, so there are no further new rounds of HIV replication (7–9). Alternatively, a “Block and Lock” approach frees infected individuals from ART by silencing HIV transcription and inducing a deep state of latency. Nevertheless, despite promising therapeutic options, the above strategies have failed to achieve significant clinical efficacy. Such failures highlight our lack of knowledge of the molecular mechanisms that govern HIV latency establishment and reversal, and the need for alternative new therapies that will be useful for eliminating the viral reservoirs (10–15). Among mechanisms that regulate the establishment of the HIV reservoir, epigenetic constraints suppress proviral gene transcription (16, 17). These are joined by low levels of basal and elongating transcription factors and the absence of the viral trans-activator of transcription (Tat), thereby ensuring suppression of that proviral transcription (18, 19). Within the infected T cells, gene transcription of the integrated provirus and the host genome are synchronized (20, 21). Both display key steps of gene transcription, which include initiation, promoter arrest, RNA polymerase pause-release, and elongation. The viral Tat protein orchestrates proviral transcription activation by binding to TAR RNA and recruiting the positive transcription elongation factor b (P-TEFb) and super elongation complex (SEC) to the viral promoter (22–26). However, despite extensive efforts to understand the mechanisms of metazoan transcriptional control and their role in the regulation of HIV gene transcription, the knowledge of how HIV latency is established is still incomplete (27).

In previous work, we have performed a whole-genome CRISPR knockout (KO) screen, documenting the enrichment of single-guide RNA (sgRNAs) in HIV-infected cells, expressing either latent or active proviral HIV (19). Our screen identified ZNF304 as a repressor of HIV gene expression. ZNF304 associates with TRIM28, thereby recruiting heterochromatin-inducing methyltransferases like the polycomb repression complex 2 (PRC2) and SETB1 to the viral promoter and silencing HIV gene expression (19). Interestingly, our CRISPR screen identified additional top hits as potential regulators of HIV gene expression—among them the activating transcription factor 1 (ATF1).

ATF1 encodes an activating transcription factor, which belongs to the ATF sub-family and bZIP (basic-region leucine zipper) family. Together with the cyclic AMP (cAMP) response element (CRE)-binding protein 1 (CREB1) and CRE modulator (CREM), ATF1 is a member of the CREB family of transcription factors that mediates key processes within cells, including proliferation, differentiation, and survival (28–30). Emerging evidence also demonstrates a role for ATF1 in modulating immune responses through regulation of T-cell proliferation and cytokine production (28). ATF1 is phosphorylated at its kinase-inducible domain on Ser63 by cAMP-dependent protein kinase A (PKA), calmodulin-dependent protein kinase I/II, mitogen, stress-activated protein kinase, and cyclin-dependent kinase 3 (cdk3) (31–34). Phosphorylated ATF1 translocates to the nucleus, where it dimerizes and forms homodimers that bind to its target sequences, such as the cAMP response element (CRE) and recruits p300/CREB-binding protein (CBP) to initiate transcription of numerous target genes (35). ATF1 phosphorylation enhances its transcriptional activity and drives cell transformation. The unique molecular structure and biological functions of ATF1 have been implicated as fusion genes in the development of human malignancy. Chromosome translocations between ATF1 and RNA-binding proteins like FUS or EWSR1 have been reported to form chimeric proteins that lead to cancer (36–38). In the context of HIV, several AP-1 binding sites within the HIV-1 LTR can bind CREB/ATF and mediate cellular activation transmitted through the cAMP-dependent PKA pathway. Previous work has also demonstrated that CREB/ATF proteins occupy the Tax responsive element (TRE) within the viral LTR. In HIV-infected cells, a sustained elevation of CREB/ATF binding activity is reported. However, the effects of ATF1 expression on HIV transcription and viral latency are not fully understood (39, 40).

In this work, we validate ATF1 as an activator of HIV gene expression and further elucidate its mechanisms of action. We demonstrate that ATF1 occupies the HIV promoter, and its expression levels are induced upon active HIV infection. ATF1 depletion decreases HIV gene expression and promotes latency establishment, both in Jurkat T cells and in human primary CD4^+^ T cells. Interestingly, ATF1 regulates CCR5 expression levels, thereby indirectly regulating HIV infection by an R5 tropic lentivirus. Mechanistically, ATF1 binds the 3′UTR of the CCR5 antisense (AS) long-coding RNA (lncRNA) and enhances its RNA transcription levels. CCR5-AS-lncRNA then binds to CCR5 mRNA and protects it from degradation by the Raly RNA-binding protein, thereby increasing protein levels of CCR5. Finally, ATF1 KO impairs the occupancy of RNA Polymerase II (RNAPII) on the HIV promoter, which is accompanied by elevated levels of H3K9me3 histone silencing marker. Genome-wide further demonstrates that ATF1 occupies cellular downstream gene promoters that indirectly may affect HIV infection.

## RESULTS

### CRISPR KO screen identifies ATF1 as an activator of HIV gene expression

We have previously reported on a whole-genome CRISPR-Cas9 knockout screen in CD4^+^ Jurkat T cells to systematically identify human host factors that potentially modulate HIV gene expression and affect latency (19, 41, 42). Cells stably expressing Cas9 were initially generated by transduction of VSV-G pseudotyped lentiviruses that drive the expression of Cas9, followed by selection with blastocidin antibiotic. A clone that stably expresses Cas9 was isolated, and cells were further propagated and then transduced with an HIV-Blue Fluorescent Protein (BFP) lentivirus, where BFP expression is regulated by the LTR HIV promoter. In this scenario, BFP expression correlates with active HIV-1 gene expression. After 2 days, cells were sorted based on their BFP expression to obtain a pure population of HIV-BFP expressing cells (BFP+). Next, cells were transduced at a low multiplicity of infection (MOI) of 0.3, with a CRISPR knockout whole-genome sgRNA library (GeCKO) (42). Subsequently, cells were subjected to puromycin selection to ensure that all surviving cells stably expressed the GeCKO library and cultured for about 1 month to allow them to establish latency—as determined by a decrease in their BFP expression (BFP−). Cells were then sorted into two groups: (i) those that do not express BFP, thus carry a transcriptionally silenced (latent) provirus [designated BFP−] and (ii) cells that expressed a transcriptionally active HIV, thus remained active [designated BFP+]. The control for this screen was cells that expressed Cas9-HIV-BFP and carried the GeCKO sgRNA library; however, harvested immediately after puromycin selection, thus should express the baseline repertoire of the sgRNA library.

**Fig 1:**
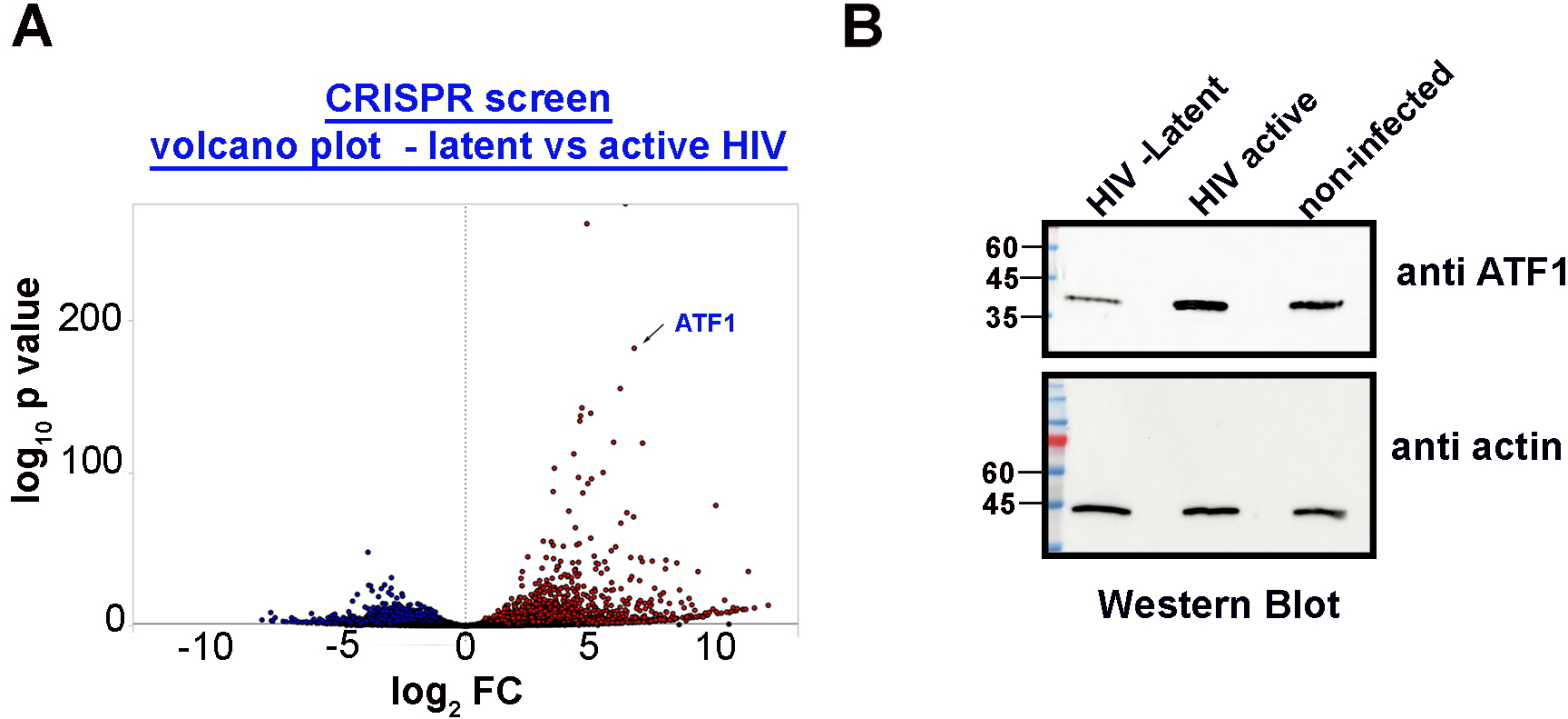
Identifying host factors that are differentially expressed in Jurkat T cells carrying active or latent proviral HIV. (**A**) Volcano plot presenting enrichment of sgRNA. Differential sgRNA enrichment is presented as log2-fold of change (FC) of normalized sgRNA counts versus −log_10_ adjusted *P* value in HIV-infected cells carrying transcriptionally latent *versus* active provirus and relative to control cells that did not go through latency establishment selection. Analysis was performed across two independent screens. Red dots are upregulated sgRNAs relative to control, while blue dots are downregulated sgRNAs relative to control. The specific ATF1 sgRNA is indicated as the top target that was enriched in latent cells. (**B**) ATF1 expression levels are elevated in cells where HIV is transcriptionally active. Jurkat T cells were transduced with HIV_GKO_ virus, and cells where HIV was either active (GFP+; mKO2+) or latent (GFP−; mKO2+) were each collected by FACS. ATF1 expression levels were analyzed using western blot. As control, non-infected cells were also monitored for endogenous levels of ATF1. Lower panel represents loading control for each indicated sample.

Twelve million cells (×100 the size of the GeCKO library) of each group were collected for genomic DNA extraction and PCR amplification of the amplicon that flanked the sgRNA and for next-generation sequencing. We applied the CRISPR analyzer algorithm to determine the enrichment of sgRNA copy counts in each of our experimental groups. Results are presented as enrichment of sgRNAs counts in latent versus active cells, relative to control infected cells (Fig. 1A). sgRNAs were ranked as upregulated or downregulated in cells where HIV is latent versus active—based on their fold of change and adjusted *P* value relative to control cells. The sgRNAs that were found to be significantly enriched were obtained from two independent screens and sequences. Among the top candidates whose sgRNA was enriched in cells that carried latent HIV was ATF1 (Fig. 1A). We next confirmed our analysis by monitoring ATF1 expression levels in cells where HIV was transcriptionally active or latent. Our western blot analysis shows that in cells where HIV latency has been established, ATF1 expression levels were low relative to cells where HIV was transcriptionally active. Moreover, ATF1 expression levels were elevated in cells where HIV was active relative to control non-infected cells (Fig. 1B).

### Modulation of ATF1 expression in Jurkat T cells

**Fig 2:**
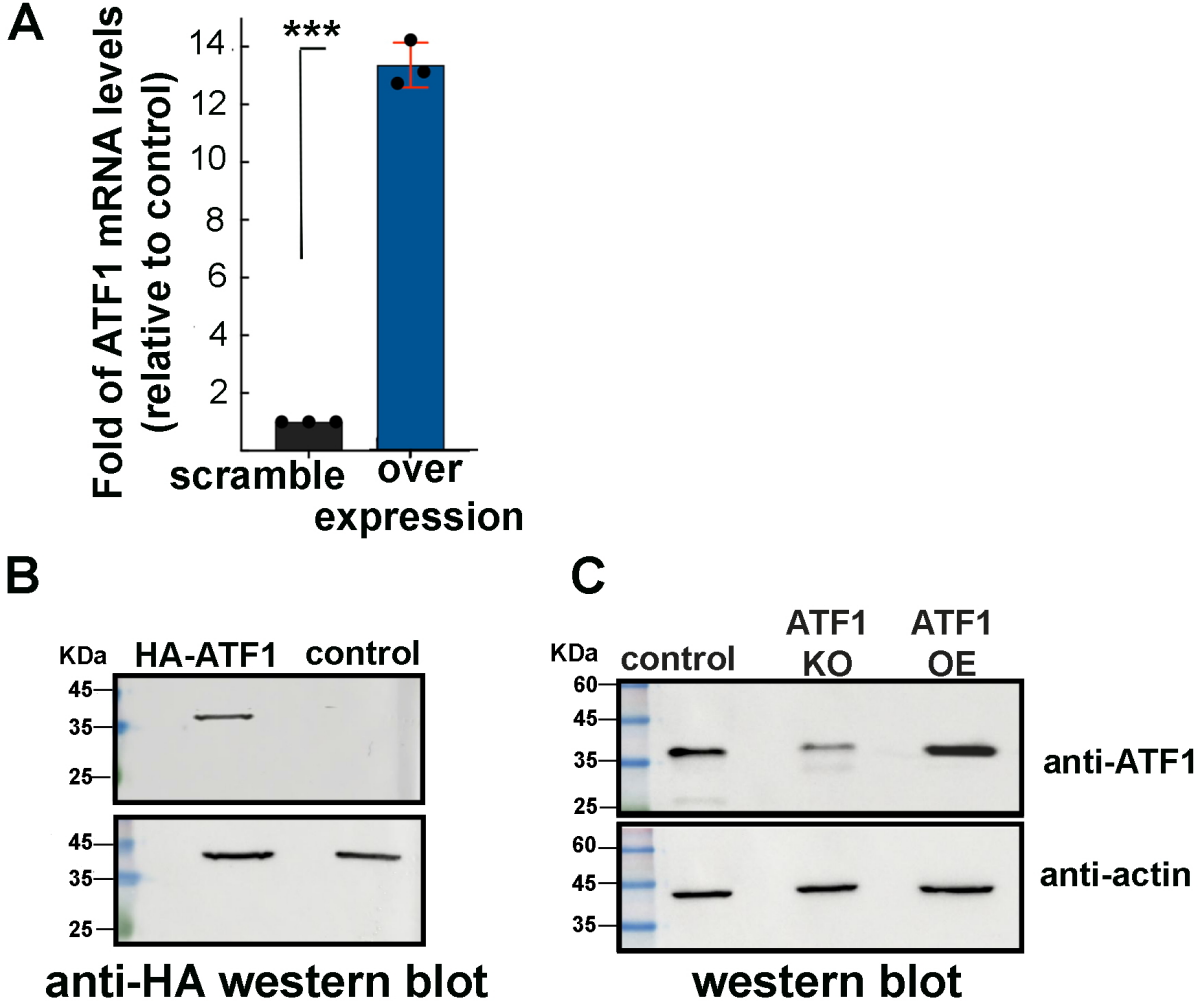
Modulation of ATF1 expression (**A**) Overexpression (OE) of HA-ATF1 in Jurkat T cells. RT-qPCR analysis monitoring relative ATF1 mRNA levels in Jurkat T cells, where HA-ATF1 expression was overexpressed (OE; blue bar). mRNA levels were normalized to *gapdh* and presented as fold of change in ATF1 mRNA levels relative to control cells expressing scrambled sgRNA set to 1 (SC; black bar). Statistical significance is based on calculating ±SD of data points from four independent experiments using two-way ANOVA. ***P ≤ 0.05. (**B**) Western blot analysis confirming expression of HA-ATF1 in ATF1 overexpressing Jurkat cells using anti-HA antibody. (**C**) Depletion of ATF1 expression. Western blot analysis using endogenous ATF1 antibody. Data confirm the depletion of ATF1 expression (KO) following treatment of cells with CRISPR-Cas9-sgRNA and HA-ATF1 OE.

We next generated Jurkat T cells, where ATF1 is either KO or over expression (OE), enabling depletion or overexpression (OE) of ATF1 expression. To achieve ATF1 OE, cells were transduced with lentiviruses that drove the expression of HA-ATF1. Following antibiotic selection, ATF1 OE was validated by RT-qPCR, exhibiting a 12-fold increase in expression levels of ATF1 mRNA (Fig. 2A; compare light blue bar vs black bar of control cells expressing scramble sgRNA). OE of HA-ATF1 was also confirmed using western blotting, using HA IgG (Fig. 2B; control cells are expressing scramble sgRNA). Depletion of ATF1 expression was achieved by transducing Jurkat T cells with lentiviruses encoding Cas9 and sgRNA that specifically targeted the ATF1 gene. Following puromycin selection, a monoclonal stable cell, where ATF1 was KO, was obtained by serial dilution. Altogether, to obtain optimal depletion of ATF1, we simultaneously introduced three sgRNAs that were positioned at different locations on the ATF1 gene. Endogenous expression of ATF1 in the single KO clone was monitored using western blot. We found that a complete KO of ATF1 expression was not obtained, and expression levels of ATF1 were only partially depleted. HA-ATF1 overexpressing cells were also validated using western blot and presented relative to control cells expressing scramble sgRNA, which was further confirmed (Fig. 2C).

### ATF1 activates HIV gene expression, which is Tat independent and inhibits latency establishment

**Fig 3:**
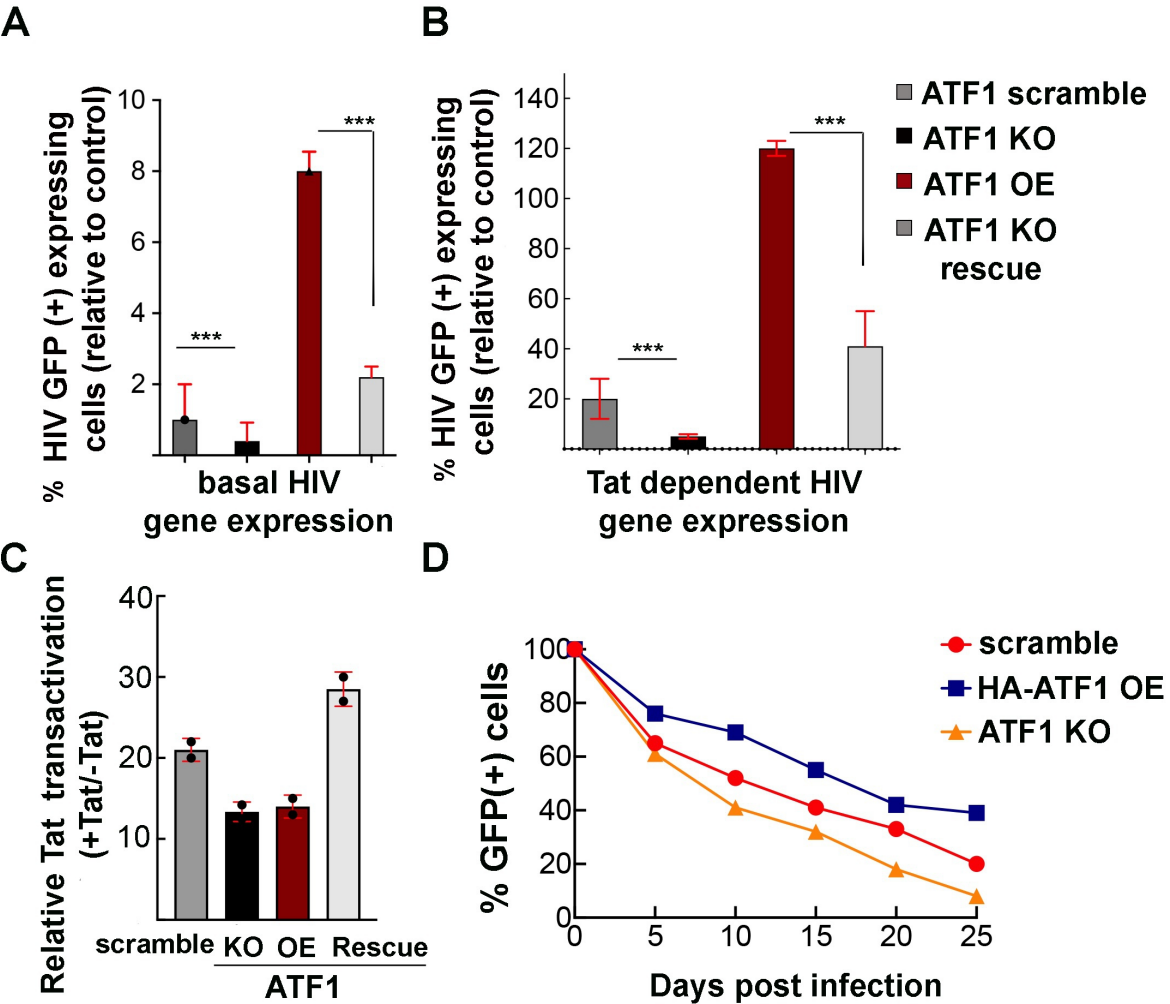
Effects of ATF1 on HIV gene expression and latency establishment. (**A**) Summary of a FACS-based analysis monitoring the relative percentage of cells that express HIV-LTR-GFP in the context of modulation of ATF1 expression levels. Control cells expressing a scrambled sgRNA (gray bar) in basal Tat-independent condition, where the percentage of GFP expression is set to 1. Cells where HA-ATF1 was overexpressed (OE; red bar); or knockout (KO; black bar); ATF1 KO cells where ATF1 expression was rescued by transducing cells with lentiviruses that drive HA-ATF1 (light gray bar). Transduction experiments were performed either with HIV-LTR-GFP for basal Tat-independent transcription (**A**); or with HIV LTR-GFP and Tat-BFP lentiviruses for Tat-dependent transcription (**B**). Statistical significance is based on calculating mean ± SD from three independent experiments using two-way ANOVA. ***P ≤ 0.05. (**C**) Bar graph presenting relative Tat transactivation (+Tat divided by −Tat). Data originated from panels (A and B) and were divided to obtain data for +Tat/−Tat values. (**D**) Kinetics of latency establishment in the context of ATF1 expression. 2D10 Jurkat T cells that carry a mini-*Tat-Rev* GFP under the regulation of the HIVLTR promoter and express either scrambled sgRNA (red), ATF1 KO (orange), or cells that overexpress HA-ATF1 (OE; blue) were stimulated with PMA/ionomycin and sorted to obtain a pure cell population that expresses GFP. Percentage of cells that express HIV GFP in ATF1 KO or OE cells was then monitored over time as a measurement of HIV latency establishment. Statistical significance is based on calculating mean ± SD from three independent experiments using two-way ANOVA. ***P ≤ 0.05 and ns: not significant.

We also conducted gain and loss-of-function experiments in Jurkat T cells to study the role of ATF1 in regulating HIV gene expression. Jurkat stable cells where ATF1 expression was modulated were transduced with an HIV-LTR-GFP lentivirus (Fig. 3). Parallel FACS-based analysis of GFP expression in HIV-infected cells, as a measure of viral gene transcription, revealed that OE of ATF1 led to an eightfold increase in basal HIV gene expression (Tat independent) relative to control cells expressing scramble sgRNA—set to 1 (compare red bar vs control gray bar). Correspondingly, depletion of ATF1 also affected HIV gene expression, leading to a fourfold decrease in basal HIV gene expression relative to control cells (compare black bar to control gray bar; Fig. 3A). Cells were then monitored for the effects of ATF1 on Tat-mediated HIV gene expression (Fig. 3B). For this, HIV-LTR GFP transduced cells where ATF1 expression was modulated were further transduced with lentiviruses that drove the expression of HIV-CMV-Tat-BFP. Our analysis confirmed that Tat-mediated HIV transcription led to a 20-fold increase above basal levels in control cells. Moreover, ATF1 OE resulted in an additional sixfold increase above observed Tat transactivation levels in control cells (Fig. 3B; compare gray control bar to red bar for cells overexpressing ATF1). ATF1 KO led to a sixfold decrease in Tat-mediated HIV-GFP expression relative to control cells expressing scramble sgRNA (Fig. 3B; compare black bar to control gray bars). We also verified HIV-GFP expression in ATF1-depleted cells following transduction of ATF1-depleted cells with lentiviruses that drove HA-ATF1 expression. Our experiments showed that introducing HA-ATF1 rescued the activation effects of ATF1 both in the absence (twofold) or presence of Tat expression (threefold above Tat transactivation in control cells) (Fig. 3A and B; light gray bars).

We then compared relative Tat transactivation in cells where ATF1 was KO, OE, or KO-rescued by HA-ATF1, by dividing readouts of HIV activation in the presence of Tat (Tat+) to those where Tat was absent (basal transactivation; Tat−) relative to control cells that expressed scrambled sgRNA. Overall relative Tat transactivation was not significantly enhanced upon expression of Tat (Fig. 3C). We therefore conclude that the effects of ATF1 on HIV gene expression are primarily Tat independent.

We further monitored the effects of ATF1 on HIV latency establishment by following the kinetics of latency establishment in the context of modulation of ATF1 expression. For these experiments, we used 2D10 cells that are latently infected Jurkat T-cell line carrying a lentiviral vector that expresses the regulatory proteins Tat and Rev *in cis* and a short-lived green fluorescent protein (d2EGFP) in place of Nef (43) (Fig. 3D). In this context, Tat carries an H13L mutation, which effectively supports HIV transcription elongation, but is attenuated, therefore inducing transcription repression (43). As expected, following T-cell stimulation of control 2D10 cells that carried scramble sgRNA, the expression of HIV-GFP was significantly induced. Control or ATF1-modulated 2D10 stimulated cells were next sorted based on their HIV-GFP expression, obtaining a pure cell population that expressed HIV-GFP. We next monitored the kinetics of latency establishment in cells where ATF1 expression was modulated by following HIV-LTR GFP by FACS (Fig. 3D). Our analysis showed that depletion (KO) of ATF1 expression induced a faster kinetic of latency establishment relative to control cells (Fig. 3D; red versus range lines). Conversely, ATF1 OE delayed the establishment of latency, as determined by the elevated levels of HIV-LTR GFP for an extended period following T-cell stimulation (Fig. 3D; blue line). These results suggest that ATF1 activates HIV gene expression and delays the establishment of viral latency.

### ATF1 activates HIV gene expression in stimulated primary CD4^+^ T cells

**Fig 4:**
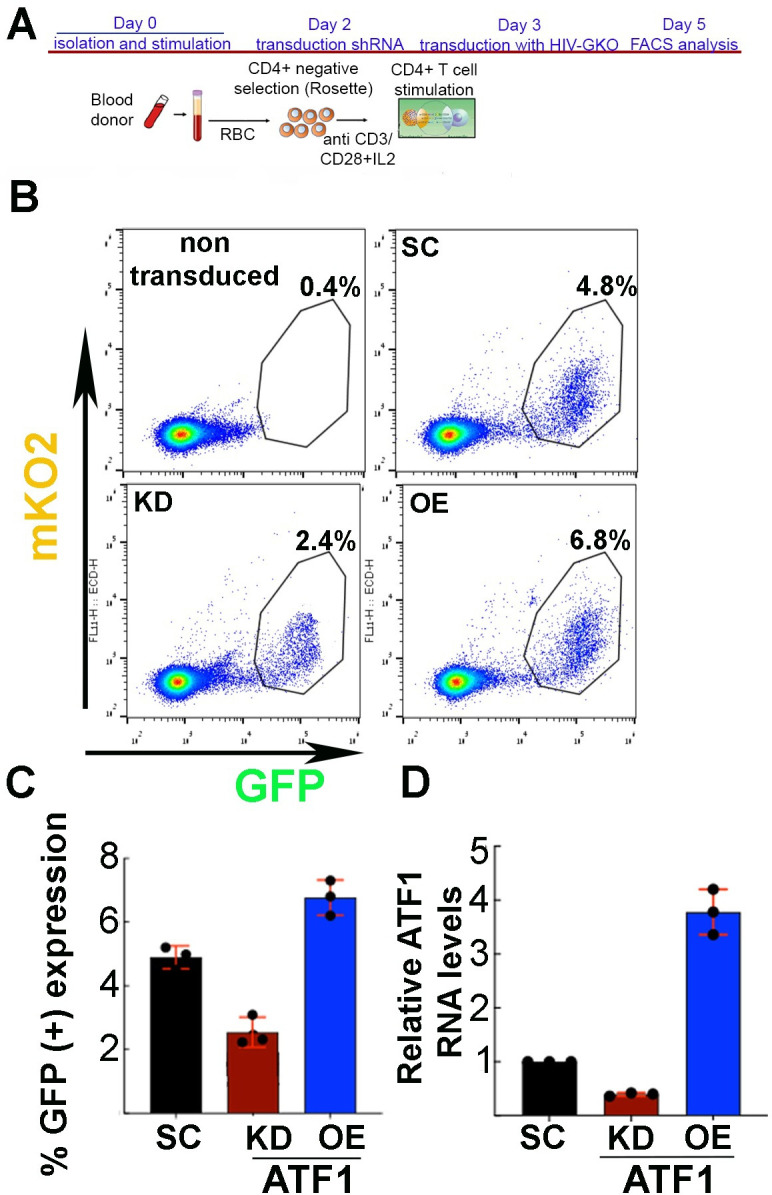
Role of ATF1 in regulating HIV infection stimulated human primary CD4^+^ T cells. (**A**) Experimental workflow for isolating primary CD4^+^ T cells and modulating ATF1 expression. Figure was generated by Biorender. (**B**) FACS analysis depicting the percentage of cells expressing HIV-LTR GFP and EF1α mKO2 in the context of modulation of ATF1 expression. Depletion of ATF1 (KD) was obtained by transducing stimulated primary CD4^+^ T cells with lentiviruses that drove ATF1-specific shRNA. ATF1 overexpression (OE) was similarly obtained by expressing HA-ATF1. Dot plots present LTR-GFP expression on the *X*-axis as a measurement of HIV gene expression and EF1α-mKO2 expression on the *Y*-axis, measuring transduction levels of live gated cells. Percentage of GFP^+^/mKO2^+^ cells is presented relative to non-transduced cells. (**C**) Quantification of the percentage of cells expressing HIV-GFP (GFP^+^) based on the above FACS analysis. Data are presented for cells expressing scramble (SC; black bar) shRNA; cells where ATF1 was depleted (KD; red bar) or overexpressed (OE; blue bar). (**D**) RT qPCR analysis confirming relative ATF1 expression mRNA levels in primary CD4 +T cells. Data were measured by RT-qPCR, normalized to *gapdh* and presented relative to cells expressing scrambled shRNA—set to 1 (black bar). Statistical significance is based on calculating mean ± SD from three independent experiments using two-way ANOVA. ***P ≤ 0.05; *n* = 3.

We then shifted our efforts for analyzing the role of ATF1 in primary CD4^+^ T cells that were isolated from healthy donors (*n* = 3) and are the natural target cells for HIV infection. Knockdown (KD) of ATF1 in primary T cells was obtained by first stimulating the cells with anti-CD3/CD28 beads and IL2 and then transducing them with lentiviruses encoding an ATF1-specific shRNA. For overexpressing HA-ATF1, stimulated cells were transduced with lentivirus expressing HA-ATF1. Lentiviruses driving the expression of scrambled shRNA were used as a control (Fig. 4A). The next day, ATF1-depleted CD4^+^ primary stimulated T cells (KD), cells expressing HA-ATF1 (OE), or control cells expressing scramble shRNA were transduced with HIV_GKO_ (a gift from Eric Verdin), which codes for a codon-optimized GFP reporter under the control of the HIV-1 promoter and in the context of expression of all viral proteins, and an mKO2 reporter under the control of a constitutive EF1αpromoter (44). HIV_GKO_ transduction was then analyzed by FACS 2 days later, parallelly monitoring transduction efficiencies (mKO2^+^), and HIV gene expression (GFP^+^; Fig. 4B).

Our results show that KD of ATF1 led to a twofold decrease in HIV-GFP gene expression, as monitored by the decrease in cells expressing HIV-LTR GFP (Fig. 4C; compare red bar to control black bar). Parallel experiments monitored the effects of HA-ATF1 OE on HIV gene expression in stimulated primary CD4^+^ T cells. Herein, OE of HA-ATF1 resulted in only a slight increase (1.5-fold) in the percentage of cells expressing HIV-GFP (Fig. 4C; compare blue bar to control black bar). mRNA levels of ATF1 in both KD and OE T cells were confirmed by RT-qPCR (Fig. 4D).

### The expression of the HIV CCR5 co-receptor correlates with CCR5-AS lncRNA levels and indirectly controls HIV infection in an ATF1-dependent manner

Multiple independent candidate gene approaches and genome-wide studies have identified clear associations between polymorphisms within or near the CCR5 gene. A well-studied example is the CCR5Δ32 mutation and its outcomes on HIV infection. Differential CCR5 regulation of expression through the expression levels of a CCR5 AS lncRNA was previously documented and found to be highly correlated with elevated HIV infection (45–47). Located upstream of CCR5-AS lncRNA, rs1015164A/G variants associate with high CCR5-AS lncRNA gene expression and correlate with high expression of CCR5. This study concluded that CCR5-AS RNA levels enhance CCR5 protein expression (45–47). Mechanistically, CCR5-AS lncRNA interferes with the binding of Raly RNA-binding protein to the CCR5-3′UTR, thereby protecting CCR5 mRNA from Raly-mediated mRNA decay and overall enhancement of CCR5 mRNA stability. Additional bioinformatic analysis indicated that while the rs1015164 SNP does not appear to carry a transcription factor binding site, an ATF1 binding site lies within an additional rs2027820 SNP located just at the first intron of CCR5-AS and is closely linked to rs1015164. ATF1 binds strongly to the rs2027820G SNP, and when linked to rs1015164A, leads to elevated CCR5-AS gene expression and increased stability of CCR5 mRNA and CCR5 expression (45–47).

**Fig 5:**
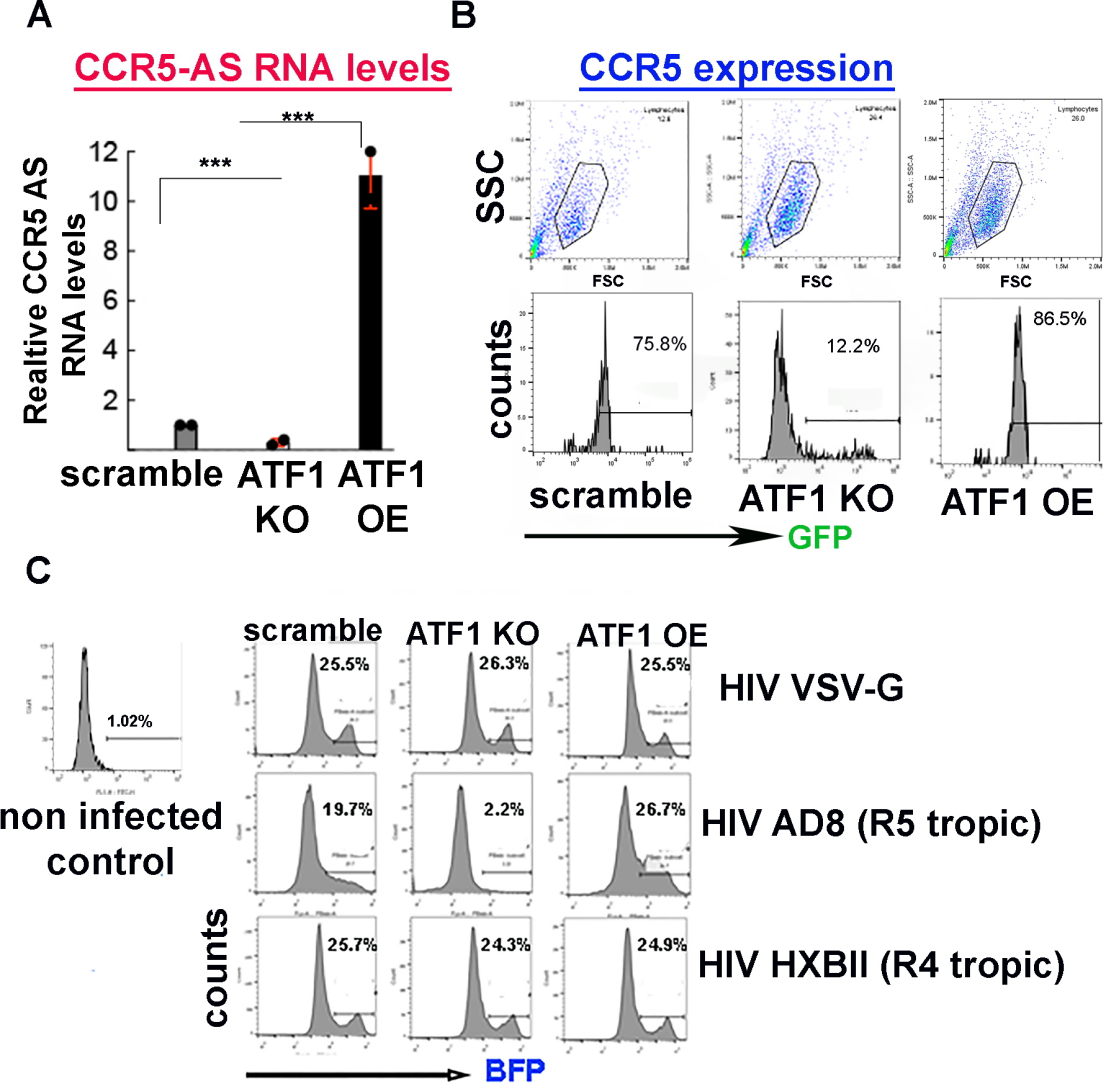
ATF1 regulates CCR5 protein expression through CCR5 anti-sense lncRNA (**A**) qPCR analysis for monitoring CCR5-anti sense (AS) RNA levels in Jurkat T cells, where ATF1 was KO or overexpressed (OE). RNA levels were normalized to *gapdh* and presented as fold of levels relative to control cells expressing scrambled sgRNA set to 1. Statistical significance is based on calculating ±SD of data points from four independent experiments using two-way ANOVA. ***P ≤ 0.05. Data are presented relative to RNA levels in cells expressing scramble sgRNA set to 1. (**B**) FACS analysis of CCR5 surface expression in Jurkat T cells where ATF1 expression is modulated. Cells, where ATF1 expression was modulated, were stained for CCR5 surface protein expression followed by staining with a secondary rabbit IgG-GFP conjugated antibody. Data are presented relative to control cells expressing scramble sgRNA. Upper panels present gated live cells, from which histograms were generated for the percentage of cells expressing HIV GFP. (**C**) ATF1 depletion of expression inhibits infection of a CCR5-tropic HIV LTR**-**Tat-BFP lentivirus pseudotyped with either VSV-G, HXBII (R4 tropic) or AD8 (R5 tropic) envelopes that were used to transduce control Jurkat T cells that express scramble sgRNA, or cells where ATF1 was KO or overexpressed (OE). Cells were analyzed for their BFP expression 48 hr post transduction and shown in the FACS histograms as percentage of cells expressing GFP, relative to non-transduced cells - set to 1%.

To extend the above observations, we sought to determine the effects of ATF1 on the surface expression of the CCR5 co-receptor, hypothesizing that low levels of ATF1 will reduce its binding to the rs2027820A SNP on CCR5-AS, thereby depleting CCR5-AS transcription and abolishing CCR5 expression. We thus monitored CCR5-AS mRNA levels by RT-qPCR in ATF1-KO or ATF1 over-expressing Jurkat T cells, comparing them to the relative mRNA levels of CCR5-AS in control cells expressing scrambled sgRNA (Fig. 5A). Our results show that relative to control cells (gray bar), KO of ATF1 significantly reduced the mRNA levels of CCR5-AS (red bar). In contrast, OE of ATF1 led to increased levels of CCR5-AS (black bar) RNA, relative to control cells (Fig. 5A). We further monitored CCR5 protein surface expression levels in ATF1 KO or OE cells by FACS, demonstrating that in KO of ATF1 cells, levels of CCR5 expression were diminished, while in the context of ATF1 OE, CCR5 expression levels were slightly elevated (Fig. 5B).

As CCR5 protein expression levels are regulated by ATF1 expression, we aimed to establish a linkage between ATF1-regulated CCR5 protein expression and HIV infection. Jurkat control cells, expressing scramble sgRNA, or cells where ATF1 expression was modulated (KO or OE), were transduced with lentivirus that drove the expression of an LTR-Tat-BFP that was pseudotyped with several HIV envelopes. Three envelopes were tested: a CCR5-tropic (R5; AD8) envelope, a CXCR4-tropic (R4; HXBII) envelope, and a control VSV-G envelope that mediates both R5 and X4 lentiviral transduction. Pseudotyped lentiviruses were then used to transduce Jurkat T cells that express both the CCR5 and CXCR4 co-receptors, where ATF1 expression was either KO or OE (Fig. 5C). We monitored the HIV-BFP expression in transduced cells by FACS, as a direct measurement for HIV infection. As expected, the VSV-G pseudotype lentiviruses efficiently transduced all the tested cells, reaching about 26% of the cells expressing HIV BFP. In contrast, lentiviruses that were pseudotyped with an AD8R5 tropic envelope were unable to transduce cells where ATF1 was depleted, and only 2% of cells expressed BFP. These AD8-pseudotyped lentiviruses successfully transduced control cells, where ATF1 was expressed. 20% of cells expressed BFP. AD8-pseudotyped lentiviruses also successfully transduced cells where ATF1 was OE. Herein, close to 40% of cells were transduced and expressed BFP. As control, R4 tropic (HXBII) lentiviruses efficiently transduced all the tested cells, and 25% of cells expressed HIV BFP (Fig. 5B).

### ATF1 regulates RNAPII recruitment to the HIV promoter and enrichment of histone H3K9me3 repressive marker

**Fig 6:**
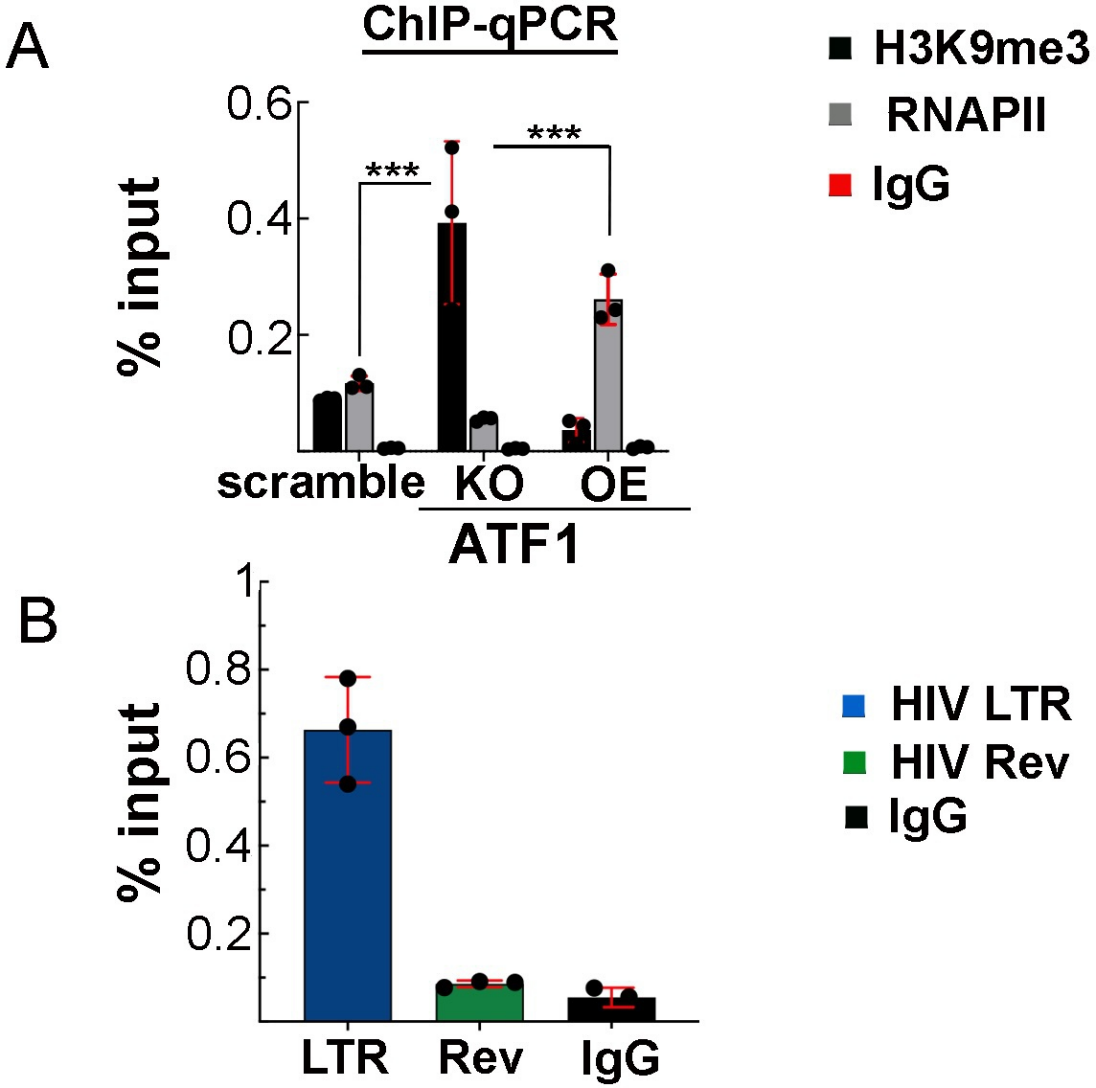
ATF1 occupies the HIV promoter and modifies the occupancy of RNA Polymerase II (RNAPII) and the levels of histone H3K9me3. (**A**) ChIP qPCR analysis in control (scramble; SC), ATF1 knockout (KO) or HA-ATF1 overexpressed (OE) 2D10 Jurkat T cells. ChIP material from cells was immune-precipitated (IP) with antibody targeting H3K9me3 repressing marker (black bars), or RNAPII (gray bars). IP fractions were analyzed for enrichment of the indicated proteins on the HIV promoter by qPCR with specific primers. Non-specific IgG served as a control (red bar). Percentage of input is means ± SD; *n* = 3; ***P ≤ 0.05 calculated between scrambled and KD cells for each antibody. n.s, not significant. (**B**) ATF1 specifically occupies the HIV promoter. ChIP qPCR analysis in 2D10 Jurkat T cells expressing HA-ATF1. ChIP material from cells was IP with HA-ATF1, anti-HA IgG, and the IP fraction was quantified by qPCR for HA-ATF1 enrichment on the HIV promoter (blue bar) or on downstream HIV *Rev* gene (green bar) with specific primers located on the NFκB sequences on the HIV promoter or at the *Rev* gene. IgG was used as control for IP (black bar). Percentage of input is means ± SD; *n* = 3; ***P ≤ 0.05 calculated between scrambled and KD cells for each antibody. n.s, not significant.

As our results indicate that ATF1 is an activator of HIV gene expression, we next sought to mechanistically explore its effects on gene expression by monitoring the occupancy of RNAPII on the HIV promoter, employing ChiP-qPCR (Fig. 6). This analysis was performed in Jurkat 2D10 cells that carry a minimal HIV *Tat-Rev-GFP* cassette under the control of the LTR promoter. Our results confirmed that in ATF1 KO cells, RNAPII occupancy levels on the viral promoter were diminished, while in cells where HA-ATF1 was OE, levels of RNAPII on the viral promoter were significantly elevated relative to control cells expressing scrambled sgRNA (Fig. 6A). Effects of ATF1 modulation of expression on the enrichment of H3K9me3 histone heterochromatin mark around the viral LTR promoter were also monitored. ChIP-qPCR analysis confirmed that relative to control cells, ATF1 KO led to a significant increase in H3K9me3 levels, while in cells where ATF1 was OE, a decrease in H3K9me3 levels around the LTR promoter was obtained.

Next, to confirm that ATF1 is indeed specifically recruited to the HIV LTR promoter, we performed ChIP qPCR on 2D10 cells that are HIV infected. For our ChIP experiments, an ATF1 antibody was not successful, and we thus used the anti HA-ATF1. Our results indicate that HA-ATF1 specifically occupies the LTR, while downstream Rev sequences exhibited low levels of ATF1 (Fig. 6B).

### Genome-wide occupancy of ATF1

**Fig 7:**
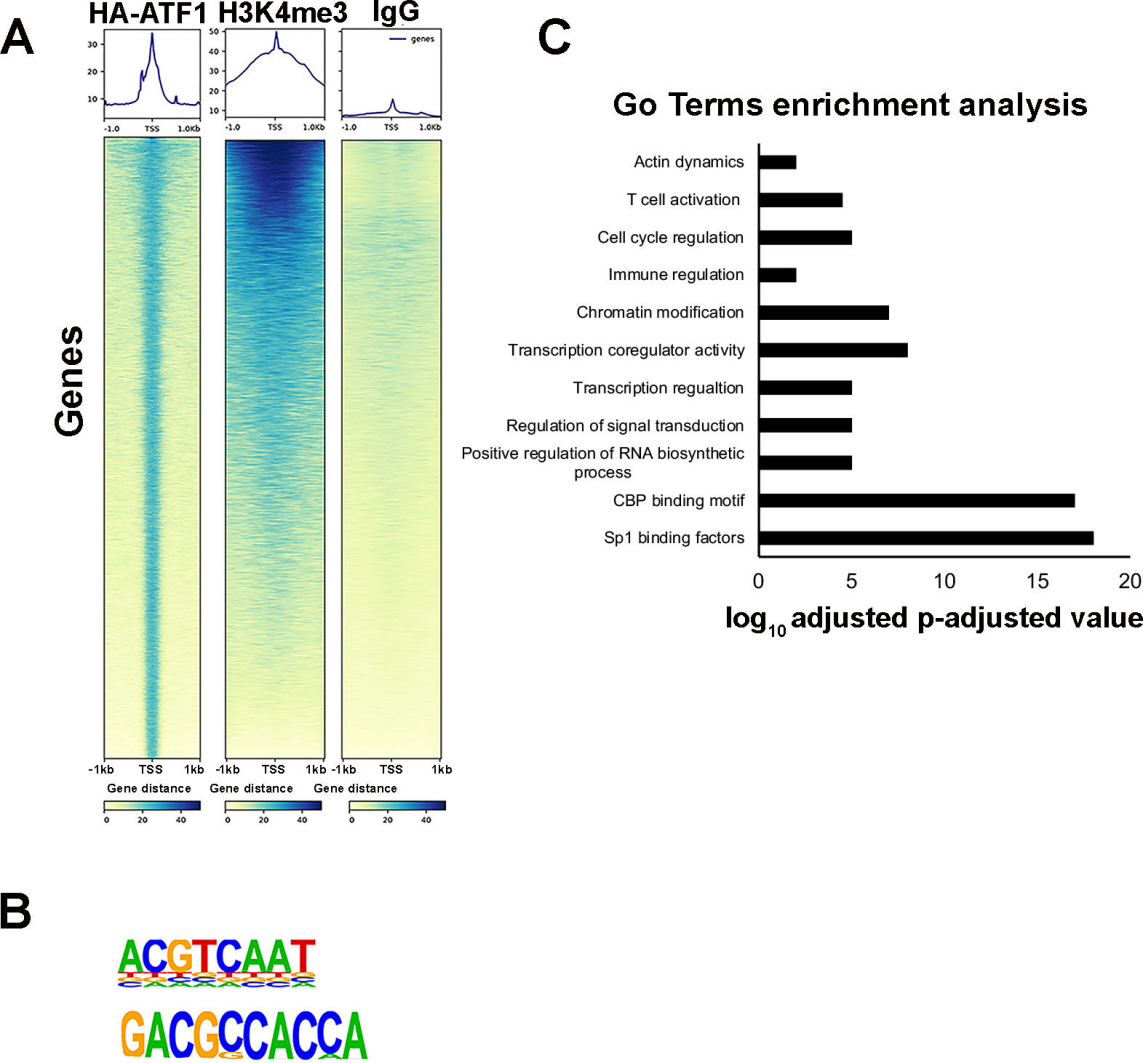
Genome-wide occupancy of ATF1. (**A**) Heatmaps presenting CUT&RUN read densities for HA-ATF1, H3K4me3, and IgG control at ±1 kb from transcription starting site (TSS). Experiments were performed in Jurkat T cells expressing HA-ATF1 using anti-HA antibody. (**B**) Consensus ATF1 binding motif on gene promoters. (**C**) GO enrichment analysis of ATF1 target genes (DAVID).

To further elucidate the regulatory role played by ATF1 in transcription activation, we mapped its genome-wide occupancy by employing cleavage under targets and release using nuclease (Cut and Run) analysis followed by high-throughput sequencing (Fig. 7A; GEO accession no. GSE286022). Our attempts to perform these experiments with an endogenous ATF1 antibody were unsuccessful. Therefore, we performed the analysis in Jurkat cells where HA-ATF1 was expressed. We used cells where HA-ATF1 protein levels were relatively low. Our analysis demonstrated that HA-ATF1 was highly enriched at gene promoters, overlapping the occupancy of H3K4me3 histone activation mark. These data suggest that ATF1 occupancy at promoters correlates with gene activation (Fig. 7A). The Cut and Run analysis also enabled us to identify the sequence motif to which the ATF1 sequence binds (Fig. 7B). Gene enrichment analysis defined the potential gene targets of ATF1, and enrichment pathways related to gene activation, transcription regulation, T-cell activation, and histone modification were identified (Fig. 7C).

## DISCUSSION

In this work, we define the ATF1 transcription factor as an activator of HIV gene expression, which plays a role in the reversal of viral latency. Previous reports indicate that downstream elements within the HIV promoter, which correspond to the AP-1 binding site, act as TRE-like cAMP responsive motifs as well as binding sites for AP-1 and AP-1-related proteins of the CREB/ATF family, thereby activating HIV gene expression through both PKC and PKA activation signals (48). Since enhanced proviral HIV-1 gene expression is associated with activation of the cAMP-dependent PKA signaling pathway, CREB/ATF can mediate cAMP/PKA activation of the HIV-1 LTR (39, 40). Our functional analysis revealed that ATF1 expression levels are elevated in cells where HIV is transcriptionally active, while in cells where HIV is transcriptionally silenced, ATF1 expression is slightly diminished relative to control non-infected cells (Fig. 1B). Furthermore, OE of ATF1 activates HIV gene expression, while its depletion suppresses viral gene expression. Significantly, following T-cell stimulation, depletion of ATF1 induces latency establishment, while its OE delays entry into latency and enhances latency reversal (Fig. 2 and 3). These observations were also confirmed in primary CD4^+^ T cells (Fig. 4). Mechanistically, we show that ATF1 binds the HIV promoter and supports the recruitment of RNAPII to the HIV promoter (Fig. 6). Changes in histone marks around the viral promoter are also observed upon modulation of ATF1 expression, implying that ATF1 is involved in the recruitment of histone modifying complexes (Fig. 6). These observations imply that ATF1 acts directly on the HIV promoter and supports activation of HIV gene expression. Interestingly, ATF1 also exerts its effects indirectly. Our studies imply that ATF1 regulates HIV gene expression through binding to the CCR5-AS lncRNA, which is correlated with higher levels of expression of the CCR5 co-receptor. CCR-AS lncRNA carries an ATF1 binding site, and upon its transcription and binding to the CCR5 3′UTR, it inhibits binding of Raly RNA protein to this region, thereby preventing CCR5 mRNA degradation. Accordingly, we demonstrate that upon ATF1 KO, levels of CCR5-AS and CCR5 expression are also diminished, and this further inhibits infection of an R5 tropic HIV (Fig. 5). Interestingly, ATF1 is also an activator of CCR5 expression itself, as its promoter carries a potential cAMP/CREB binding site, thereby affecting endogenous *CCR5* transcription (49). Coupling this suggested pathway to the fact that ATF1 binds directly to the HIV promoter and, in addition, enhances CCR5 protein expression via the ATF1-CCR5-AS lncRNA axis points to a novel regulatory network that overall supports HIV infection. Not surprisingly, our CUT&RUN analysis shows that ATF1 occupies gene promoters, thereby regulating other downstream gene targets. These are currently being investigated for potential indirect effects on HIV gene expression. Of note, our experiments were performed in cells that stably express HA-ATF1. We are aware of the potential interference between the endogenous ATF1 protein and the transient HA-ATF1 one. However, we could not find a suitable ATF1 antibody to successfully execute the analysis.

Proteins of the ATF1 family play a key role in cellular response to multiple stresses, including altered metabolic conditions, anoxia and hypoxia, redox stress, and virus infection. Linking extracellular stress signals to intracellular transcriptional programs, ATF proteins are known to regulate the expression of genes that promote cell survival or initiate programmed cell death (apoptosis) pathways. These proteins are important for homeostasis, and their dysregulation may promote disease progression, including cancer (50). However, a role in HIV gene expression regulation and viral latency remains unexplored. Among ATF proteins, ATF4, a key transcription regulator of the integrated stress response (ISR), an evolutionarily conserved intra-cellular signaling network that coordinates cell responses to various environmental and pathological stress (51), has been previously associated with HIV infection, as it was identified as a factor that is upregulated during HIV-1 infection, thereby activating HIV replication and enhancing both Tat-independent and Tat-dependent viral gene expression (50, 52). Its effects were detected both in cell line models and primary T cells (50, 53), and defined both as direct via binding to the viral promoter, or indirect via ATF4-mediated signaling pathways (54). The latter includes the cellular ISR pathway, the mitochondrial unfolded protein response (UPRmt), and the IFN signaling pathway—all associated with cell stress and viral infection and are presumably targeted during HIV infection (54). As our observations indicate that ATF1 modulates HIV gene expression both directly by binding the HIV promoter and indirectly via CCR5 expression regulation, we can speculate that similarly to AFT4, ATF1 potentially modulates specific signaling pathways, thus indirectly enhances HIV infection. In this context, both ATF1 and ATF4 can be defined as potential LRAs that can reactivate HIV-1, thereby serving as potential agents that might be integrated in clinically “Shock and Kill” therapeutic approaches for eliminating the viral reservoir (55). Alongside, inhibition of ATF1 may induce a deep state of latency and thereby be used as part of a “Block and Lock” approach.

As recent studies have shown that ATF1 is OE in several cancers, including hepatocellular carcinoma, non-small cell lung cancer, esophageal squamous cell carcinoma, and cervical cancer, specific inhibitors for ATF1 are being developed. One example is an aryl antimonate compound, P6981, which inhibits DNA binding of ATF1, thereby affecting the growth of patient-derived malignant cells that express the chimeric protein EWS-ATF1 (28). It will be interesting to explore the effects of such specific drugs and similar ones in inducing HIV latency in people living with HIV under therapy regimen. Other currently used ATF1 modulators that act on ATF1-upstream pathways may also be tested. JS1287, for example, modulates ATF1 mRNA levels, ERK1/2 expression, and reduces the release of inflammatory cytokines IL-6, IL-12, and IL-17A (56). An additional novel dual-target inhibitor of RSK1/MSK2, APIO-EE-07, is used against colon cancer and inhibits cell proliferation through the downregulation of ATF1 expression and increased expression of Bax, caspase-3, and PARP. Other drugs can modulate post-translation modifications of ATF1, primarily an arginine methyltransferase (PRMT) 5, which enhances EWSR1-ATF1-mediated gene transcription and maintains SU-CCS-1 cell growth. A clinical-stage PRMT5 inhibitor, JNJ-64619178, inhibits cell proliferation by affecting ATF1 demethylation. Although these drugs may be potentially toxic to some extent, their contribution to eliminating the infected reservoir or inducing deep latency is worthwhile testing in future clinical trials.

## MATERIALS AND METHODS

### Cells

Jurkat T cells are immortalized human T lymphocytes that are used for studying HIV infection. 2D10 is a Jurkat-based latency T cells model, carrying a mini-HIV cassette coding for an H13L mutated *Tat* and *Rev* and a 2dGFP reporter gene all regulated under the control of the LTR promoter. Cells were maintained in RMPI medium (GIBCO) supplemented with 10% fetal bovine serum, 2 mg/mL l-glutamine, penicillin-streptomycin, and non-essential amino acids (Sigma-M7145). Human embryonic kidney HEK293T cells were used to generate pseudotyped viral particles and were maintained in DMEM complete medium (GIBCO). All cells were cultured at 37°C with 5% CO_2_.

### Isolation of primary CD4^+^ T cells

Human CD4^+^ T lymphocytes were isolated from buffy coats from anonymous healthy donors and obtained from the Soroka Medical Center Hospital Blood Bank. PBMCs were isolated over a Ficoll gradient (Millipore) and were maintained at 2 × 10^6^ cells/mL overnight at 37°C. The following day, CD4^+^ T cells were isolated by negative selection with the RosetteSepTM Human CD4^+^ T-Cell Enrichment Cocktail, resulting in homogenous populations of CD4^+^ T cells with a purity of 90–95% as monitored by flow cytometry. Resting CD4^+^ T cells were cultured in complete RPMI media containing recombinant human IL2 at 20 U/mL (Roche) to a final concentration of 10^6^ cells/mL and then stimulated using anti-CD3/CD28 Dynabeads (Invitrogen). Levels of activation were monitored by FACS, measuring staining with APC anti-human CD25 (Bio-Legend 302609) and Pacific Blue anti-human CD69 (Bio-Legend 310919). Stimulated cells were counted, centrifuged for 5 min at 1,500 rpm and resuspended in fresh RPMI and IL-2 at a final concentration of 0.5 × 10^6^ cell/mL before transduction with high-titer HIV_GKO_ lentivirus at an MOI of 10. Transduced cells were cultured for an additional 48 h in complete RPMI media containing recombinant human IL2 and dynabeads at a ratio of 25 µL human beads per 10 million cells, before being analyzed by FACS for transduction levels.

### Antibodies

The following antibodies were used: anti-ATF1 (ab181569), anti-HA (ab9110), and anti-actin. For ChIP-qPCR, to detect histone marks activation markers, we used anti-H3K4me3 (number 9751S) and anti-Rab 1 (RNAPII) (4H8) from Cell Signaling Technology. For CCR5 surface expression, an anti-CCR5 polyclonal antibody was used (ab277802).

### CRISPR screen using the GeCKO library

A whole genome CRISPR-KO screen was performed in HIV-infected Jurkat T cells for the identification of host factors that control HIV gene expression, as described by Kransnopolsky et al. (19). Jurkat T cells that stably express Cas9-Flag were generated by lentiviral transduction followed by blastocidin antibiotic selection. A Cas9 stable clone was then transduced with pseudotyped lentivirus that expressed HIV-LTR-BFP, and cells were sorted by FACS, based on their BFP expression at 72 h post-transduction, thus obtaining a pure cell population that expressed BFP. Thereafter, 1.3 × 10^8^ cells were transduced with a lentivirus that expressed the GeCKO sgRNA library (42). GeCKO library was a generous gift from the laboratory of Ophir Shalem and consists of multiple sgRNA guides that target a total of 19,052 human genes. For each gene, there are 6 sgRNA constructs, along with 1,864 sgRNAs against miRNA with 4 constructs each, making a total library of 122,417 sgRNA members. The library also contains control sgRNA that does not target any gene (42). Cell transduction of lentivirus that drives the GeCKO library was performed at an MOI of 0.3 to statistically ensure the integration of a single sgRNA per cell. Following transduction, cells were subjected to puromycin selection to obtain stable cells that expressed both Cas9 and the sgRNA GeCKO library. All cells carried integrated transcriptionally active HIV (BFP+). GeCKO-expressing cells were then grown over a period of 30–40 days, allowing them to establish latency, as determined by FACS that monitored the decrease of LTR-BFP expression. At this point, cells (40 × 10^6^ GeCKO-expressed cells − × 300 of library size of 1.3 × 10^5^ members) carrying either transcriptionally active (BFP+) or transcriptionally latent HIV (BFP−) were sorted based on their BFP expression. 12 × 10^6^ (×100 above library size) cells from each group were collected for sgRNA PCR-amplicon amplification and NGS sequencing (primers carried the suitable barcodes for NGS). As the experimental baseline control for sgRNA enrichment, GeCKO sgRNA-infected cells were isolated right after GeCKO lentiviral transduction, thus were not allowed to establish latency and expressed the entire GeCKO library repertoire. sgRNA enrichment was analyzed by the CRISPR analyzer, comparing sgRNA normalized counts for each experimental group of cells relative to the control cells. A volcano enrichment plot was generated, presenting upregulated or downregulated sgRNAs relative to control cells in cells that carry latent versus active cells. ATF1 sgRNA was enriched in latent cells, indicating that it is an activator of transcription.

### Generation of pseudotyped lentivirus

Pseudotyped lentiviruses were generated in HEK293T cells as described (19). Briefly, the transgene plasmid was transfected into cells using CaCl_2_ together with additional lentiviral packaging plasmids coding for *Gag*, *Pol*, *Tat*, *Rev*, and the VSV-G envelope. Transfections were performed in a 10 cm format. The supernatant containing the virus was harvested 72 h post-transfection, filtered through a 0.45 µm filter spun at 2,000 rpm for 5 min to remove cell debris and stored at −80°C. For transduction, 2 × 10^5^ Jurkat T cells were transduced with the pseudotyped particles in a 24-well plate format. After 16 h, the medium containing lentiviral particles was changed. Following transduction, cells were cultured in a medium supplemented with 2 µg/mL of puromycin or blastocidin to eliminate non-transduced cells. Upon the death of all the control cells, the medium was changed, and surviving cells were propagated for future experiments. Where indicated, additional R4 or R5 tropic envelopes replaced the VSV-G expression plasmid. For transducing CD4^+^ primary T cells, we used HIV_GKO_ (a gift from Eric Verdin), which codes for a codon-optimized GFP reporter under the control of the HIV-1 promoter and in the context of expression of all viral proteins, and a mKO2 reporter under the control of the constitutive promoter EF1α (44).

### Modulation of ATF1 expression in Jurkat cells and in stimulated primary CD4^+^ T cells

KO of ATF1 expression in Jurkat cells or primary CD4^+^ T cells was achieved by transducing cells with lentivirus that express Cas9/sgRNA specifically targeting ATF1. Cells were next selected on puromycin, and a polyclonal cell population was obtained and propagated. Monoclonal cells, where ATF1 expression was KO, were obtained by serial dilution, and ATF1 expression was verified using western blot using endogenous ATF1 antibody. To achieve ATF1 OE, cells were transduced with lentiviruses that drove the expression of HA-ATF1. Following antibiotic selection, clonal-resistant cells were subjected to RT-qPCR to confirm ATF1 OE. Western blot analysis also confirmed HA-ATF1 OE using either endogenous ATF1 antibody or HA antibody.

To obtain ATF1 knockdown or OE in stimulated primary CD4^+^ T cells, cells were isolated from healthy donors (*n* = 3) and stimulated with anti-CD3/CD28 beads (1:1 ratio of beads-to-cell number). Cells were cultured on stimulation media (RPMI + IL2), and at day 2 post-isolation, cells were transduced with lentivirus expressing shRNA against ATF1 or with lentivirus expressing HA-ATF1. The next day (day 3), cells were transduced with HIV_GKO_. At 48 h post-transduction (day 5), cells were analyzed by FACS for HIV-GFP and mKO_2_ expression.

### Latency establishment

To monitor the effects of ATF1 in the regulation of HIV latency, we followed the kinetics of latency establishment of 2D10 cells, which are Jurkat T cells that are used for studying latency and carry a minimal mini virus coding to H13L *Tat*, *Rev*, and a *d2EGFP* reporter cassette under the regulation of the HIV LTR promoter (43). ATF1 KO was achieved with lentivirus that drove Cas9-sgRNA and targeted ATF1. ATF1 OE was obtained with lentivirus expressing HA-ATF1. Control 2D10 cells expressed scrambled sgRNA. Cells were stimulated with PMA/ionomycin, which are widely used for T-cell stimulation and induce HIV gene expression. Cells were then sorted by FACS to isolate those that express active HIV GFP. Cells were further grown, during which their HIV GFP expression was followed by FACS.

### CCR5 surface expression and transduction with R4 or R5 tropic lentiviruses

Jurkat cells were stained for CCR5 surface expression by FACS with a CCR5 antibody followed by stain with a secondary rabbit IgG-GFP control. LTR BFP-Tat lentiviruses were pseudotyped with AD8 or HXBII envelopes as described above.

### Chromatin immunoprecipitation and qPCR (ChIP qPCR)

2D10 cells expressing scramble sgRNA or cells where ATF1 expression was depleted (KO) were cross-linked with 1% formaldehyde for 10 min and then washed with PBS and reverse cross-linked with glycine (125 mM; 5 min). Cells were then lysed for 10 min on ice in 130 µL sonication buffer (20 mM Tris [pH 7.8], 2 mM EDTA, 0.5% SDS, 0.5 mM phenylmethylsulfonyl fluoride, and 1% protease inhibitor cocktail), and the nuclear pellets were collected. DNA was fragmented by sonication at the following settings: amplitude 20% for 30 cycles at 10 s on/10 s off. Samples were centrifuged (15 min, 14,000 rpm, 4°C). The soluble chromatin fraction (25 µg) was collected and immunoprecipitated (IP) overnight at 4°C on a rotating wheel in IP buffer (0.5% Triton X-100, 2 mM EDTA, 20 mM Tris [pH 7.8], 150 mM NaCl, and 10% glycerol) with 2.5 µg of one of the indicated antibodies (ATF1; H3K9me3; RNAPII). The next day, the IP material was incubated with 25 µL dynabeads protein G for 2 h to ensure the binding of the antibody to the magnetic beads. DNA was eluted with freshly prepared elution solution (1% SDS and 0.1 M NaHCO_3_) and heated at 65*°*C overnight to reverse-crosslink the samples. Precipitated DNA fragments were then extracted using a ChIP DNA clean and concentrator kit (ZYMO Research), and HIV DNA levels were quantified by qPCR with the primers specifically located on the NFκB region at the HIV-LTR promoter. All signals were normalized relative to input DNA. ChIP assays were also performed with an anti-rabbit or mouse IgG as negative control.

### Cleavage under targets and release using nuclease

Jurkat T cells where HA-ATF1 was stably expressed were harvested and resuspended in nuclear extraction buffer (20 mM 4-[2-hydroxyethyl]−1-piperazineethanesulfonic acid [pH 7.9], 10 mM potassium chloride, 0.1% Triton X-100, 20% glycerol, and 0.5 mM spermidine) supplemented with complete EDTA-free protease inhibitor cocktail (Roche) for 10 min on ice. Nuclei were then spun at 600 × *g* for 3 min at 4°C, resuspended in fresh nuclear extraction buffer, and subjected to cleavage under targets and release using nuclease (CUT&RUN) analysis using the CUT&RUN Kit (EpiCypher). Nuclei were attached to concanavalin A beads and permeabilized with digitonin, and aliquots of 5 × 10^5^ nuclei were incubated with anti-HA (Abcam), anti-H3K4me3 (Cell Signaling Technology; number 9751S; 1:100) and anti-IgG (EpiCypher; 1:100) on nutator overnight at 4°C. The next day, nuclei were washed two times with Digitonin buffer (EpiCypher), incubated with pAG-MNase (EpiCypher) for 10 min at room temperature, and washed two more times. Next, MNase was activated with 1 mM calcium chloride for 2 h at 4°C. The reaction was stopped by adding stop buffer supplemented with *Escherichia coli* spike-in DNA (EpiCypher). Libraries were constructed following a standard protocol for the CUT&RUN Library Prep Kit (EpiCypher). Constructed CUT&RUN libraries were sequenced on an Illumina NovaSeq X platform, obtaining 150 nt paired-end reads.

### Primers used for qPCR analysis

#### Primers on the HIV promoter

NFκB forward: 5′-AGGTTTGACAGCCGCCTA-3′

NFκB reverse: 5′-AGAGACCCAGTACAGGCAAAA-3′

gapdh forward: 5′-AGCCACATCGCTCAGACAC-3′

gapdh reverse: 5′-GCCCAAACGACCAAATCC-3′

### Statistical measurements

Statistical evaluation was performed with GraphPad Prism 7 using two-way ANOVA with no correction for multiple comparisons. Number of independent data points refers to biological replicates. Each data point, as mentioned in the figure legends, represents the mean of three to four independent experiments with the errors calculated based on mean ± SD. Differences were considered statistically significant and denoted as ***P ≤ 0.05; n.s., not significant.

## Data Availability

The analysis was carried out using the NeatSeq-Flow platform (57). Raw sequencing data in FASTQ format were trimmed by Trim Galore (version 0.4.5) (length = 25, q- 25), and reads that were too short were discarded. FastQC, version 0.12.0, was used for read quality control. BWA Mapper (version 0.7.12, default parameters t = 20, B = 5) (58) was used to align paired-end reads to reference human genome assembly hg38 and to the spike-in control (*Escherichia coli*) reference genome assembly. SAMtools (59) was used to remove PCR duplicates and create coordinate-sorted BAM files. Normalization between samples and IgG was performed with a custom script that calculates read scaling factor based on spike-in DNA reads of each sample. The scaling factor was then used in the bamCoverage to normalize each sample according to its spike-in. Bigwig files were created by bamCoverage function from deepTools (version 1.5.91) (60), and reads mapped to blacklisted areas designated by ENCODE (61) were filtered. Reads that refer to off-chromosome locations were removed with BedClip (version 377) and sorted by Bedtools (version 2.30.0) (62). Bigwig files were then created with bedGraphToBigWig by deepTools (60). ATF1 peaks were called by SEACR (version 1.3), the top 1% of enriched regions in target data were selected, and peaks 100 bp and less apart were merged. Peaks were viewed in Integrated Genomics Viewer (version 2.16.1) (and annotated with Homer (version 4.11. Peaks 500 bp upstream or downstream from the TSS were selected for further analyses. Matrices were generated with DeepTools createMatrix, and Heatmaps were plotted with deepTools plotHeatmap (60). Raw and processed data were submitted to the GEO under accession no. GSE286022.
